# Effect of different proportions of glycerol and D-mannitol as plasticizer on the properties of extruded corn starch

**DOI:** 10.3389/fnut.2023.1335812

**Published:** 2024-01-17

**Authors:** Xin Xu, Bin Wang, Wei Gao, Jie Sui, Jianfei Wang, Bo Cui

**Affiliations:** ^1^State Key Laboratory of Biobased Material and Green Papermaking, Qilu University of Technology, Shandong Academy of Sciences, Jinan, China; ^2^School of Food Science and Engineering, Qilu University of Technology, Shandong Academy of Sciences, Jinan, Shandong, China; ^3^Department of Food Science and Engineering, Shandong Agricultural University, Taian, China; ^4^Shandong Academy of Agricultural Sciences, Jinan, Shandong, China

**Keywords:** corn starch, glycerol, D-mannitol, plasticizing, twin-screw extrusion, physicochemical properties

## Abstract

In this study, thermoplastic starch (TPS) was prepared by melt extrusion process, in which glycerol and/or D-mannitol were used as plasticizers, and the effect of different glycerol/D-mannitol ratios (4:0, 3:1, 2:2, 1:3, and 0:4) on the physicochemical properties of the extruded starch samples was investigated. The short-range molecular order, crystallization, gelatinization, thermal stability, and thermal properties of the TPS samples were analyzed through attenuated total reflection-Fourier transform infrared (ATR-FTIR) spectroscopy, X-ray diffraction (XRD), rapid visco analysis (RVA), differential scanning calorimetry (DSC), and thermogravimetric analysis (TGA). The results showed that the crystallinity and short-range molecular order of the TPS samples increased with increasing glycerol content. Conversely, the water absorption index (WAI) and water solubility index (WSI) of the TPS samples decreased with increasing glycerol content. In addition, the TPS samples with higher glycerol content exhibited higher gel and thermal stabilities. This study provides a theoretical basis for starch extrusion and plasticization in the preparation of TPS-based materials with specific properties.

## 1 Introduction

With an increase in the global population, the demand for petroleum-based materials is also increasing (1). The misuse of these materials causes serious harm to human health, marine life, and the environment on which humans depend (2–4). In addition, the non-renewable nature of oil, as a raw material for plastic production, has led to severe shortage of resources and rising oil prices (5). Recently, biopolymers have attracted extensive attention because of their abundance and biodegradability (6). Fossil fuel materials are highly resistant to physical, chemical and biological factors, and their structure and molecular size limit their biodegradation performance (7). However, the composition of biopolymers makes them easily broken down by enzymes or by water, carbon dioxide and microorganisms in the soil, making them biodegradable (8). Starch is one of the most abundant and readily available polysaccharides in plants. It is readily available, renewable, biodegradable, and can be used to produce environmentally friendly materials. Therefore, starch presents a significant opportunity for utilization in the synthesis of biodegradable materials (9). However, as a polysaccharide polymer compound, starch contains a large number of hydroxyl groups in the molecule, and can easily form strong hydrogen bonds between and within the molecules. Therefore, its inability to undergo thermoplastic processing severely restricts its potential applications (10). Because the melting temperature of starch is higher than its degradation temperature (11), it is necessary to destroy the crystal structure of pure starch, reduce its glass transition temperature, and enhance its flexibility and processability by adding low molecular weight compounds, such as water or glycerin (12, 13).

Thermoplastic starch (TPS) is prepared by applying thermal and shear stresses in the presence of a plasticizer and limited amount of water. Thermal processes with mechanical forces decrease the interaction between the starch chains and increase the movement of amylose and amylopectin chains (14). Compared with starch, TPS is abundantly available, renewable, and completely biodegradable, which makes it highly promising for a wide range of applications (15). However, water-based TPS has disadvantages such as easy aging, embrittlement, and strong hydrophilicity (16). Therefore, selecting an appropriate plasticizer is a feasible and important method to overcome these disadvantages. According to the literature, plasticizers such as glycerol (17), sorbitol (18), xylitol (10), citric acid (19), formamide (20), and urea (21) have a good plasticizing effect. Glycerol is the most commonly used polyol plasticizer. It consists of three hydroxyl functional groups that can form a wide range of intermolecular and intramolecular hydrogen bonds; thus, the starch system is better plasticized under heat and shear stress (22, 23). D-mannitol is an acyclic sugar alcohol with poor hygroscopic properties and excellent thermodynamic stability (De (24)). The difference in the molecular structures of glycerol and D-mannitol leads to different plasticizing effects on TPS. Therefore, it is important to investigate the influence of co-plasticizing glycerol and D-mannitol on the properties of TPS (25).

At present, numerous studies have focused on utilizing different plasticizer mixtures to improve the properties of TPS-based materials (26, 27). Yang et al. investigated the properties of thermoplastic starch films from different plant sources (28). Foret et al. studied the differences in functional properties between cannabis tablets and wheat TPS co-extruded materials at different concentrations (29). These studies focus on the differences in the properties of starch-based materials. However, it is equally important to determine the structure and properties of TPS before it is made into a material. Moreover, the effects of adding two plasticizers, glycerol and D-mannitol, on the structure and physicochemical properties of TPS are rarely studied in detail. Although there are many types of starch, corn starch is the main source of starch. In addition, corn starch is widely used in the research of starch-based materials because of its low price, convenient availability and excellent physical and chemical properties (30). Therefore, corn starch was used as the raw material in this study, and the amount of fixed plasticizer was 20% of the mass of the corn starch. The short-range molecular ordering, thermal degradation stability, and crystallization, thermal, and gelatinization properties of the extruded TPS samples plasticized with different proportions of glycerol and D-mannitol (4:0, 3:1, 2:2, 1:3, and 0:4) were studied. Exploring the effect of plasticizer on the structure and properties of thermoplastic starch is helpful to understand the plasticizer’s plasticizing mechanism during starch extrusion. The properties of thermoplastic starch affect the functional properties of starch-based materials. This study will provide theoretical basis and research direction for the subsequent preparation of thermoplastic starch-based materials with specific functions.

## 2 Materials and methods

### 2.1 Materials

Corn starch, with 13.20% moisture and 27.53% amylose content, was purchased from Shandong Longli Biotechnology Co., Ltd. (Shandong, China). Glycerol (relative molecular weight: 92.09, purity ≥99.0%) was provided by Tianjin Fuyu Fine Chemical Co. Ltd. (Tianjin, China). D-mannitol (molecular weight: 182.17, purity: 96.0–101.5%) was purchased from Beijing Solarbio Science and Technology Co., Ltd. (Beijing, China).

### 2.2 Preparation of extruded samples

Before extrusion, the corn starch and plasticizer were evenly mixed. Briefly, 500 g of corn starch (based on wet weight), 100 g of purified water, and a constant weight of 100 g of plasticizer (G + D = 100 g, where glycerol and D-mannitol represent G and D, respectively) were weighed and thoroughly mixed at 25°C using a high-speed mixer (1015XL, Shenzhen Sanlida Electric Co., Ltd., Guangdong, China). Different proportions of glycerin and D-mannitol plasticizers (4:0, 3:1, 2:2, 1:3, and 0:4) were added to the mixture to obtain five different blends. Each mixture was sealed in a polythene zip-lock bag, marked, and placed at 25°C for 24 h.

An SHJ20 laboratory co-directional twin-screw extruder (Nanjing Giant Machinery Co., Ltd., Nanjing, China) was used for melt blending. The extrusion temperature of each zone was set to 60, 70, 100, and 90°C, the screw speed was 10 Hz, and the feed rate was 8 Hz. After cooling, the extrudates were cut into segments of 1 cm and baked in an oven (DHG-9035A, Shanghai Yiheng Scientific Instrument Co., Ltd., Shanghai, China) at 40°C for 48 h. The extrudates were crushed separately at room temperature and passed through an 80-mesh sieve to obtain extruded corn starch samples (The samples were symbolized with Sx, in which x is the G/D ratio of 4/0, 3/1, 2/2, 1/3 and 0/4, respectively. The samples were named S4/0, S3/1, S2/2, S1/3, and S0/4), which were encapsulated in a zip-lock bag and placed in a desiccator (JB-BLGZQ, Jiangsu Runhong science and education Equipment Co., Ltd., Jiangsu, China) for testing.

### 2.3 Moisture content

The moisture content of the starch samples was determined using a MA 45 quartz infrared moisture analyzer (Sartorius, Germany). First, spread 2.5 g sample evenly on aluminum sample pans and then heated to 110°C for 10 min. Finally, the moisture content of the samples was measured directly using a moisture analyzer.

### 2.4 Water absorption index and water solubility index

The absorbability and water solubility of the as-prepared starch samples were determined according to a previously described method (31) with minor modifications. First, the beaker and centrifuge tubes were washed and dried to a constant weight. Then, 1 g of the sample and 12 mL of deionized water were evenly mixed and stirred in a water bath at 30°C for 30 min. Centrifugation was performed at 5000 rpm for 25 min. The supernatant was collected and dried at 105°C to a constant weight, and the precipitate was dried at 60°C to a constant weight. Water absorption index (WAI) and water solubility index (WSI) were calculated using Eqs. (1) and (2).

(1) $$WAI=\frac{M_{1}-M_{2}}{M_{1}}\times 100$$

(2) $$WSI=\frac{M_{3}}{M_{0}}\times 100$$

where M_0_ is the weight of the corn starch sample (1 g), M_1_ is the weight of the precipitate after centrifugation (g), M_2_ is the weight of the precipitate after complete drying (g), and M_3_ is the weight of the soluble material in the supernatant (g).

The experiment was repeated thrice and the average value was calculated.

### 2.5 X-ray diffraction

A Rigaku X-ray diffractometer (SmartLab SE, Japan) was used to scan the corn starch samples to obtain their X-ray diffraction (XRD) patterns. Samples were scanned at diffraction angles (2θ) of 5–40° at a step size of 0.02°. The scan rate was 10 °/min. The voltage and current were set to 40 kV and 40 mA, respectively. The XRD patterns were analyzed using the MDI Jade 6.0 software (Materials Data, Inc., USA). The relative crystallinity (RC) of each sample was calculated using Eq. (3).

(3) $$\begin{array}{l} \mathrm{Relative}\;\mathrm{crystallinity}\;\left(RC\right)\\ =\frac{\mathrm{Crystalline}\;\mathrm{area}}{\mathrm{Total}\;\mathrm{area}\;\left(\mathrm{amorphous}+\mathrm{crystalline}\right)}\; \end{array}$$

### 2.6 Attenuated total reflection-Fourier transform infrared spectroscopy (ATR-FTIR)

The functional groups and structural changes in the extruded samples (with varying ratios of glycerol and D-mannitol as plasticizers) were analyzed using a Nicolet iS10 spectrometer (Thermo Fisher Scientific, USA). The sample was evenly laid on the surface of the diamond crystal. The test parameters were as follows: the spectrum of air recorded as the background, scan range of 4,000–500 cm^−1^, resolution of 4 cm^−1^, and a total of 32 scans. The deconvolution spectrum of each sample was analyzed from 1,200 to 800 cm^−1^, and the absorbance ratio of each sample was calculated at 1047/1022 (degree of double helix order, DO) and 995/1022 (degree of double helix, DD) (32).

### 2.7 Pasting properties

The gelatinization characteristics of the starch samples were determined using a rapid viscosity analyzer (RVA-4, Perten Instruments, Australia). The test parameters were similar to those reported in a previous study (33), but with a few modifications. First, 3 g of sample was mixed with 25 mL of distilled water in an aluminum pan. The specific heating procedure is as follows: (1) maintaining the temperature at 50°C for 50 s and then heating to 95°C at 10°C/min; (2) maintaining the temperature at 95°C for 2.5 min, then reducing it to 50°C at 10°C/min and finally, maintaining it at 50°C for 2 min. The blades were rotated at 960 rpm for the first 10 s, and then at 160 rpm for the rest of the test.

### 2.8 Differential scanning calorimetry

The thermal characteristics of the samples were determined using a differential scanning calorimetry (DSC) 214 Polyma calorimeter (NETZSCH, Germany). Approximately 2 mg of sample was weighed and evenly mixed with 6 μL of purified water (sucked using a pipette) in an aluminum crucible, which was then properly sealed. The samples were incubated at 25°C for 24 h to balance the moisture. The sample test procedure included heating from 20 to 150°C at a heating rate of 10°C/min. Thermodynamic parameters (initial temperature T_o_, peak temperature T_p_, termination temperature T_c_, and enthalpy change ΔH) and DSC exothermic curves were recorded and analyzed.

### 2.9 Thermogravimetric analysis

The thermal stabilities of the extruded corn starch samples were determined using a thermogravimetric analysis (TGA) 2 (LF) thermogravimetric analyzer (Mettler Toledo, Switzerland). Approximately 15 mg of the extruded starch sample was placed in a ceramic crucible. The samples were tested by heating at a rate of 10°C/min under N_2_ atmosphere at temperatures ranging from 30°C to 600°C. A thermodynamic degradation curve was derived and the TG curve was obtained by derivative thermogravimetric analysis (DTG). The TG and DTG curves were used to determine the relationship between the degradation rate of the extruded starch samples and the temperature.

### 2.10 Statistical analyses

All the experimental indicators were measured at least in triplicate. The data were analyzed using the Statistical Product and Service Solutions (SPSS) software, version 23.0 (IBM SPSS Statistics, USA). One-way analysis of variance (ANOVA) and Tukey’s test were used to determine the significance level (*p* < 0.05), and the results were recorded as mean value ± standard deviation. Graphs were obtained using Origin 2021 (OriginLab Corp., USA).

## 3 Results and discussion

### 3.1 Moisture content

**Figure 1:**
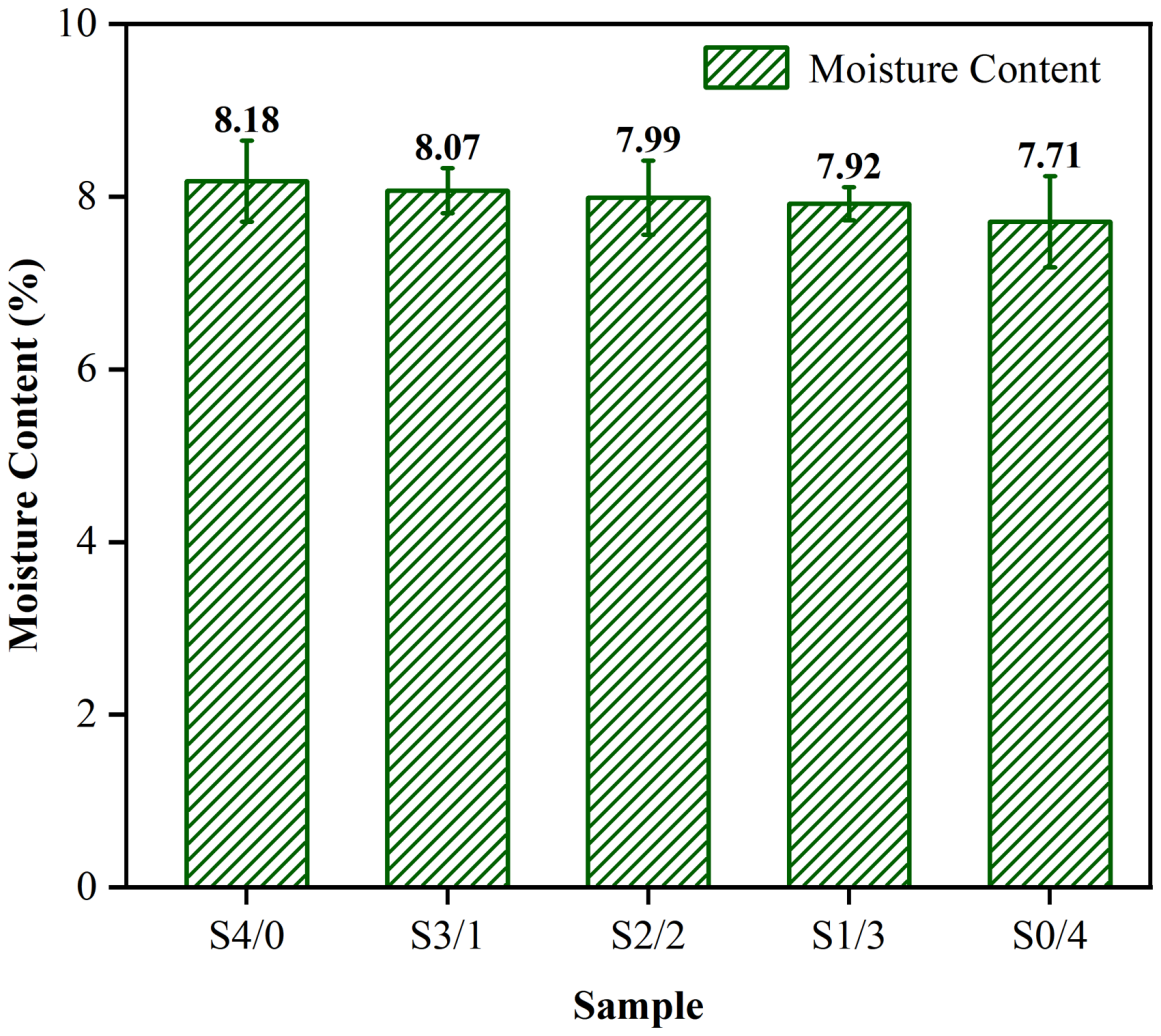
Moisture content of TPS samples plasticized with different proportions of glycerol and D-mannitol. The samples were symbolized with Sx, in which x is the G/D ratio of 4/0, 3/1, 2/2, 1/3, and 0/4, respectively. ^a^G and D represent glycerol and D-mannitol contents, respectively.

Glycerol is often employed as a plasticizer in starch-based systems due to its ability to form strong hydrogen bonds with starch molecules, increase the hydrophilicity of the system by introducing additional hydroxyl groups, and exhibit a high affinity for water (34). The moisture content of the TPS samples prepared with a constant total weight of plasticizer and changing G/D ratios is shown in Figure 1. The moisture content of the samples increased with increasing glycerol content. Figure 1 shows the increase in the moisture content from 7.71 ± 0.53% to 8.18 ± 0.47%. However, there was no significant difference between the moisture content of the samples (*p >* 0.05). Therefore, the differences in performance between different samples is almost unaffected by the moisture content of the samples.

### 3.2 WAI and WSI

**Figure 2:**
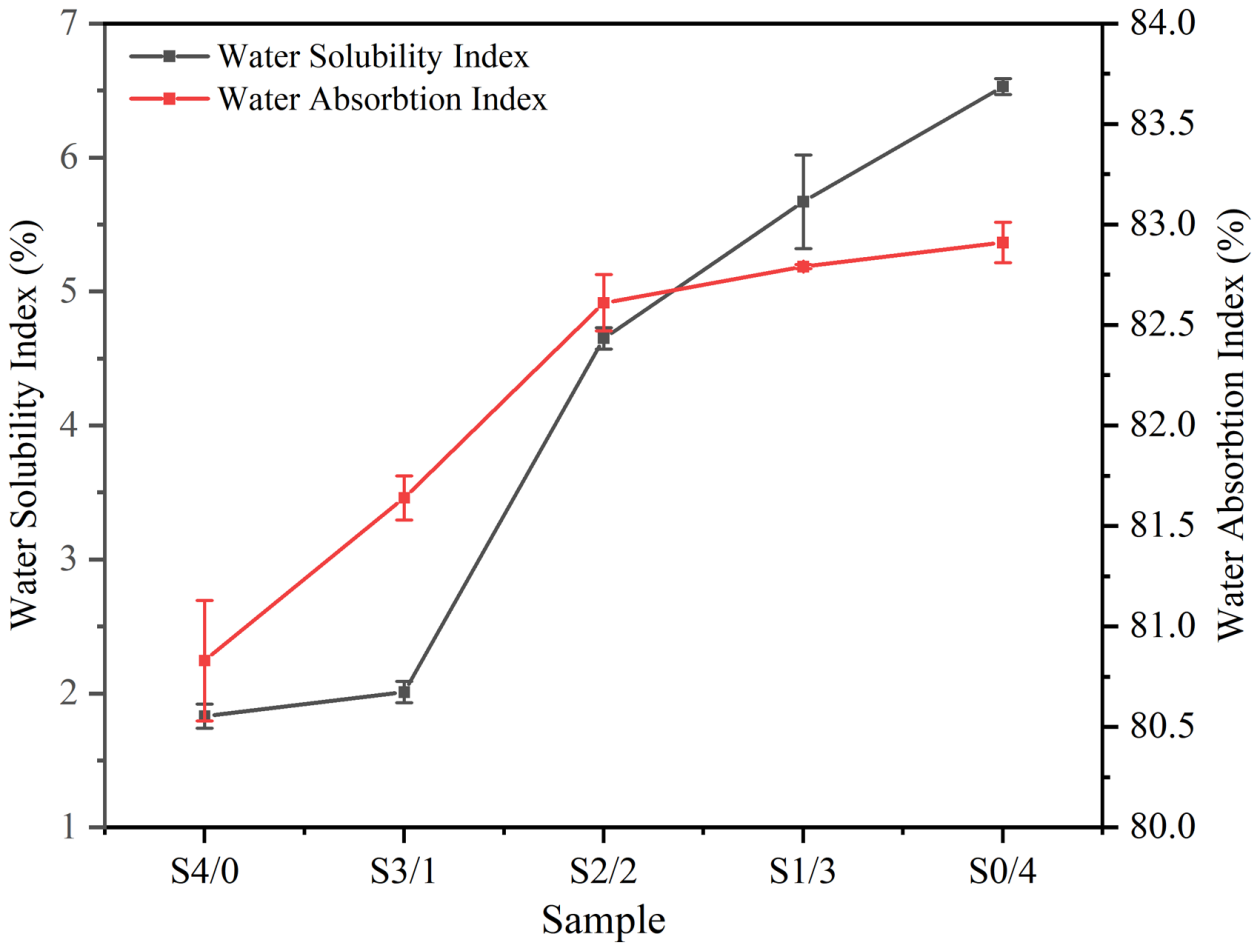
Water absorbtion index and water solubility index of TPS samples plasticized with different proportions of glycerol and D-mannitol.

Water sensitivity is an important criterion for practical applications of TPS. TPS plasticized with only water is highly hydrophilic, and its humidity sensitivity limits its mechanical properties and affects its applications. Figure 2 shows the WSI and WAI of TPS plasticized with different proportions of glycerol and D-mannitol. WAI represents the strength of the water absorption capacity of the sample, which depends on the availability of hydrophilic groups and the ability of macromolecules to form gels (35). All samples exhibited a high WAI, which may be related to the gelatinization of starch after extrusion and also to the degradation of starch molecules, resulting in an increased hydroxyl exposure (36). Starch gelatinization causes hydrogen bond breakage, and more hydroxyl groups are exposed and combined with water molecules to form hydrogen bonds, resulting in an increased water absorption by the TPS samples (35).

Glycerol has a lower molecular weight (G: *M*_r_ = 92.09) than D-mannitol (D: *M*_r_ = 182.17). The small glycerol molecules are more likely to enter the starch chain to form hydrogen bonds, forming a denser membrane network structure and reducing water absorption (26, 37). The TPS water solubility index increased from 1.83 ± 0.09% to 6.53 ± 0.06% with decreasing glycerol content. Plasticizers can promote plasticizer-polymer interactions by reducing the interactions between the biopolymer chains (38). Glycerol has a low molecular weight and straight chains that can easily interact with starch molecules (5). However, the insertion of large molecules of D-mannitol between the starch chains is typically hindered, which then easily escape into the solution (39). This behavior is similar to that reported for sweet potato starch films (40).

### 3.3 Analysis of crystalline properties

**Figure 3:**
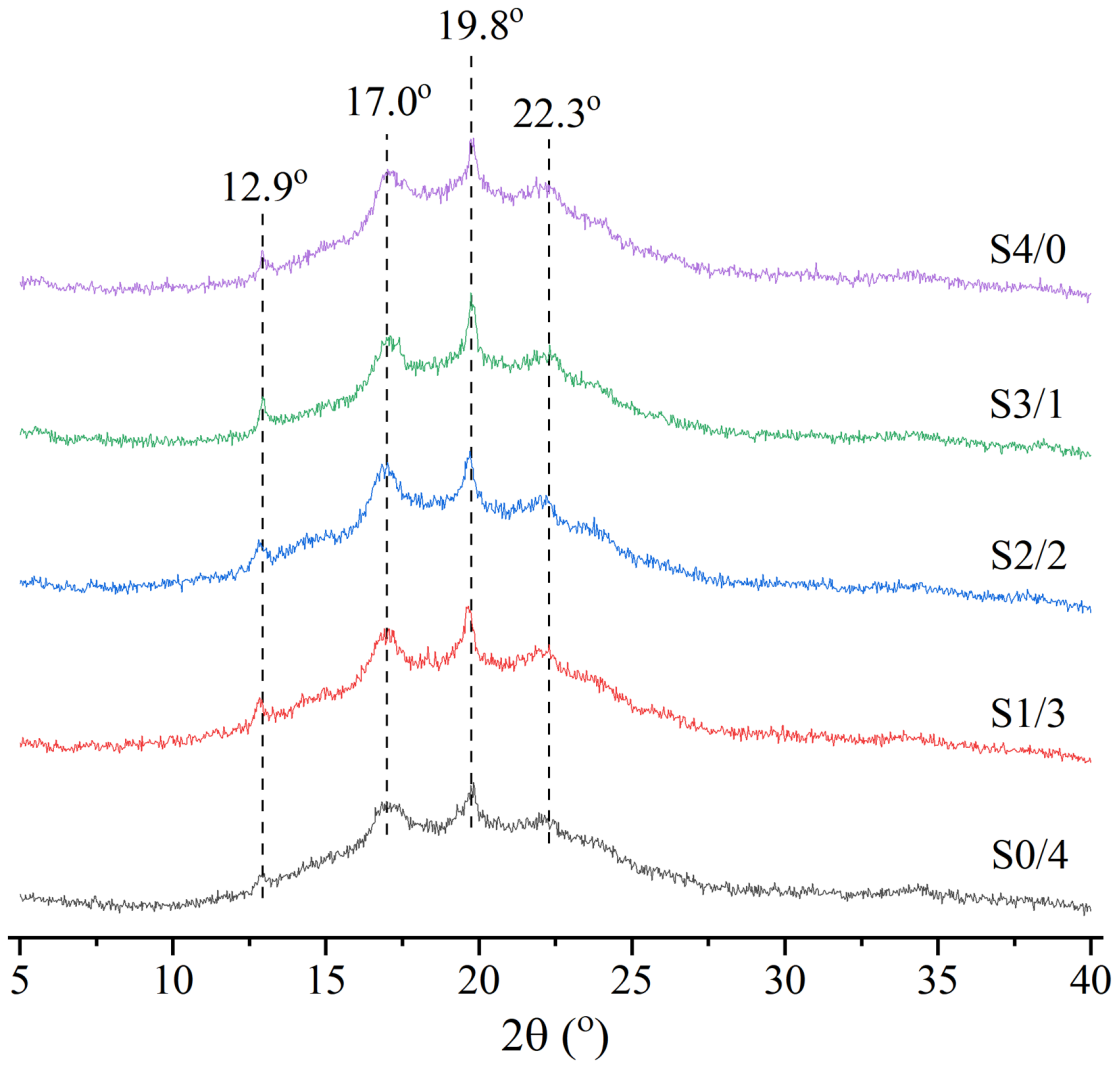
XRD analysis of TPS samples plasticized with different proportions of glycerol and D-mannitol.

Figure 3 shows the XRD patterns of the extruded TPS samples with different plasticizer G/D ratios. According to the literature, natural corn starch has an A-type crystalline structure and the crystallization peaks appear at 2θ = 15°, 17°, 18°, and 23° (30). Figure 3 shows that the peak locations of the TPS samples are 2θ = 12.9°, 17.0°, 19.8°, and 22.3°, belonging to the B + V crystal structure (27). Gao et al. reported that extrusion blow-molded films, co-molded with glycerol and water, have B-type (diffraction peaks at 5.6°, 17°, 22°, and 24°) and V-type (diffraction peaks at 13° and 19.8°) crystal structures (41). Similar crystallization peaks were observed in this study. The formation of V-shaped crystals may be related to amylose-glycerol interactions (42).

The RC values of the TPS samples calculated using the MDI Jade 6.0 software are shown in Table 1. Among all the samples, S4/0 exhibited the highest diffraction peak intensity and RC value. Table 1 shows that the RC values of the samples increased gradually with increasing specific gravity of glycerol. The strong hygroscopicity of glycerol can easily produce backflow and recrystallize the TPS samples (22). Yoksan & Dang have reported that free glycerol can migrate into biodegradable polymers to facilitate their mobility and eventual crystallization (43). Compared to D-mannitol, glycerol has a lower molecular weight and can enter starch molecules more easily to form hydrogen bonds. D-mannitol has a large molecular weight. Therefore, only a small amount can enter the starch molecules, making it difficult to open the molecular chains to form a regular arrangement. With an increase in the glycerol content, the fluidity of the starch chains was enhanced, and the ordered structure of the starch molecules rearranged to form microcrystals. Therefore, the RC values increased with increasing glycerol content in the TPS samples. The result is consistent with Liu et al., the plasticizer with small molecular weight is subjected to small steric hindrance when entering starch polymer, and it is easier to form hydrogen bond and uniform network structure with starch, thus improving crystallinity (39).

**Table 1:** Relative crystallinity and short range ordered structure of TPS samples plasticized with different proportions of glycerol and D-mannitol.

Samples	Relative Crystallinity (%)	Degree of double helix order (1,047/1022)	Double helix degree (995/1022)
S4/0	9.99 ± 0.98^a^	1.36 ± 0.09^a^	1.19 ± 0.04^a^
S3/1	9.92 ± 0.76^a^	1.30 ± 0.11^ab^	1.16 ± 0.07^ab^
S2/2	9.76 ± 0.31^ab^	1.23 ± 0.06^ab^	1.11 ± 0.02^ab^
S1/3	9.02 ± 0.32^ab^	1.19 ± 0.04^ab^	1.10 ± 0.02^ab^
S0/4	8.66 ± 0.30^b^	1.15 ± 0.01^b^	1.07 ± 0.00^b^

Values are expressed as mean ± standard deviation of multiple replicated samples. Values in the same column with different letters indicate significant differences (*p* < 0.05). The samples were symbolized with Sx, in which x is the G/D ratio of 4/0, 3/1, 2/2, 1/3, and 0/4, respectively. ^a^G and D represent glycerol and D-mannitol contents, respectively.

### 3.4 FTIR spectroscopic analysis

**Figure 4:**
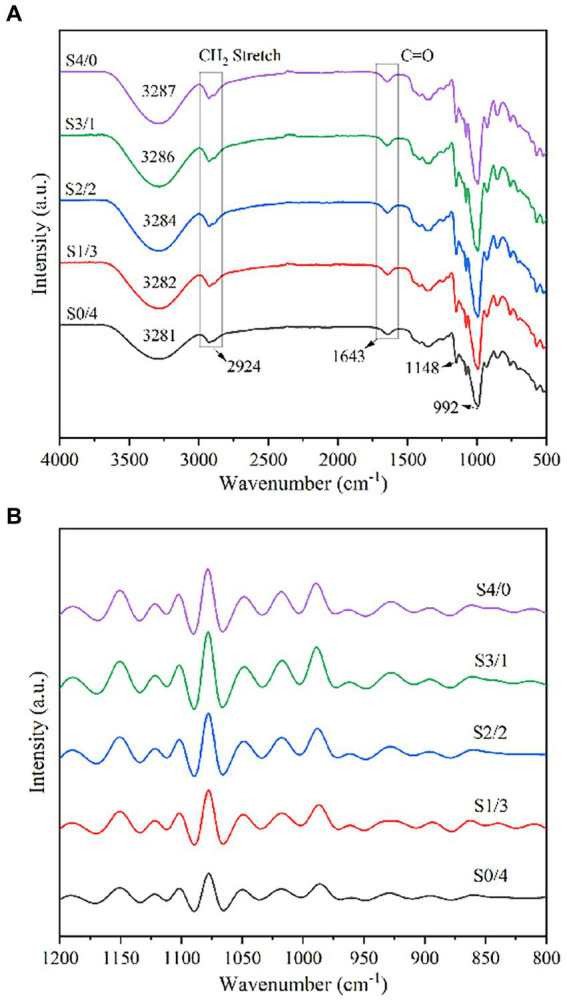
Attenuated total reflection-Fourier transform infrared spectroscopy of TPS samples plasticized with different proportions of glycerol and D-mannitol.

FTIR spectroscopy was used to analyze the functional groups and structural changes in the samples. The FTIR spectra of the TPS samples with different plasticizer G/D ratios are shown in Figure 4A. The characteristic peak of the TPS samples at approximately 3,280 cm^−1^ can be attributed to the hydrogen bonds formed between the starch polymer chains and plasticizer. Similar spikes were reported in some previous studies (44, 45). With increasing D-mannitol content, the characteristic peak at 3280 cm^−1^ gradually shifted to a lower wavenumber, indicating that the -OH groups of the starch molecules formed more stable hydrogen bonds with D-mannitol. Lozano-Navarro et al. reported that non-covalent interactions may occur between the functional groups of chitosan, starch, glycerol, and natural extracts (46). The peak at approximately 2,920 cm^−1^ can be attributed to the asymmetric stretching vibration of -CH_2_ (44). The characteristic absorption peak of the carbon-based tensile vibration (-C=O) was observed at 1650–1640 cm^−1^ (47). The characteristic peaks at approximately 1,148 cm^−1^ and 993 cm^−1^ can be attributed to C-O-H and C-O-C stretching vibrations, respectively (48, 46). With an increase in the D-mannitol content, the characteristic peaks at 1150 cm^−1^ and 990 cm^−1^ remained almost unchanged, indicating that glycerol and D-mannitol can form a small number of weak hydrogen bonds at the C-O position of C-O-H groups of starch and the C-O position of C-O-C groups, and that the bond energies to form these hydrogen bonds are similar.

Figure 4B shows the deconvolution spectra of the TPS samples with different G/D ratios in the 1,200–800 cm^−1^ region. According to the calculation results for the intensity ratios of 1047/1022 and 995/1022, the DO and DD values corresponding to the TPS samples are listed in Table 1. With increasing glycerol content, the values of DO and DD showed an increasing trend, corresponding to the crystallization properties. The double helix is formed primarily depends on the hydrogen bonds. When the total amount of plasticizer is constant, increasing the concentration of glycerol can improve the mobility of molecular chains of starch, resulting in the formation of new hydrogen bonds between the starch molecules and glycerol. Therefore, increasing the glycerol content can improve the molecular order of the TPS samples (49).

### 3.5 Analysis of pasting properties

The gelatinization performance is closely related to the function of starch and is critical for the application of starch-based products (50). Table 2 lists the peak viscosity (PV), trough viscosity (TV), breakdown viscosity (BDV), final viscosity (FV), and setback viscosity (SBV) of all the TPS samples. With increasing D-mannitol content in the plasticizer blend, the PV, TV, BDV, FV, and SBV values for all the samples first decreased and then increased. The high PV indicates that the molecular structure of starch is destroyed after heat and high shear stress treatments, the long chain molecules of starch undergo shortening, and an effective molecular interaction between the plasticizer and starch polymer occurs (51). Generally, BDV reflects the stability of starch gel. The results indicate that TPS plasticized with only glycerol or D-mannitol demonstrates a higher gel stability. Both the FV and SBV are associated with short-term regeneration. The low FV and SBV of the starch-glycerol-D-mannitol blend system could be due to the interaction between the plasticizer and starch, which interferes with the rearrangement of starch molecular chains. These results echo the long-range and short-range ordered results of the samples. Glycerol is more likely to form hydrogen bond interaction with starch molecules, which is manifested as an increase in viscosity of the TPS paste. The TPS sample with only D-mannitol as the plasticizer showed higher PV, TV, BDV, FV, and SBV than those with the mixed plasticizers, possibly because of the competition between D-mannitol and starch for water and the entanglement of amylose (52).

**Table 2:** Pasting properties of TPS samples plasticized with different proportions of glycerol and D-mannitol.

Samples	Peak Viscosity (Pa·s)	Trough Viscosity (Pa·s)	Breakdown Viscosity (Pa·s)	Final Viscosity (Pa·s)	Setback Viscosity (Pa·s)
S4/0	0.462 ± 0.083^a^	0.363 ± 0.062^a^	0.099 ± 0.024^a^	0.863 ± 0.119^a^	0.500 ± 0.064^a^
S3/1	0.258 ± 0.009^bc^	0.198 ± 0.006^bc^	0.060 ± 0.004^b^	0.587 ± 0.016^bc^	0.389 ± 0.011^b^
S2/2	0.242 ± 0.018^bc^	0.185 ± 0.012^c^	0.057 ± 0.006^bc^	0.562 ± 0.036^c^	0.377 ± 0.024^b^
S1/3	0.187 ± 0.039^c^	0.153 ± 0.029^c^	0.034 ± 0.009^c^	0.403 ± 0.077^d^	0.250 ± 0.048^c^
S0/4	0.350 ± 0.087^b^	0.274 ± 0.076^b^	0.076 ± 0.012^ab^	0.727 ± 0.105^ab^	0.453 ± 0.035^ab^

Values are expressed as mean ± standard deviation of multiple replicated samples. Values in the same column with different letters (a–d) indicate significant differences (*p* < 0.05).

### 3.6 Analysis of thermal properties

**Figure 5:**
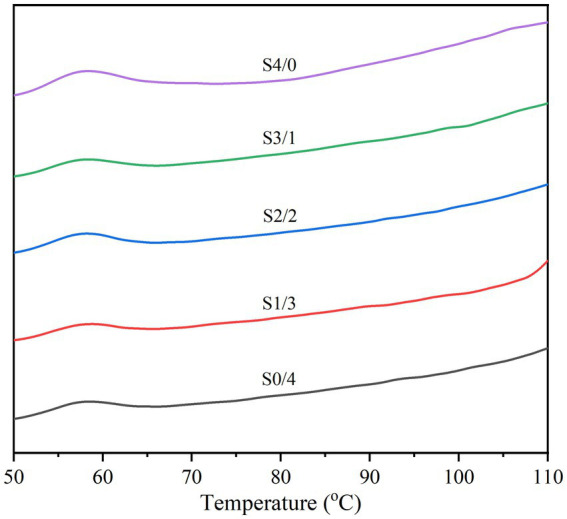
Thermal properties of TPS samples plasticized with different proportions of glycerol and D-mannitol.

Figure 5 and Table 3 show the thermodynamic diagram, thermal transition temperatures (T_o_, T_p_, and T_c_), and the enthalpy of gelatinization (ΔH) for all the TPS samples. Changes in the crystal melting and double helix structure during the heating of starch molecules were detected by DSC, and the thermal properties of the TPS samples were analyzed. All TPS samples showed small gelatinization peaks and enthalpies, indicating secondary gelatinization of starch. A possible reason for this is that the sample is revived during storage, forming a new and less stable double-helical ordered structure (53). In addition, Table 3 clearly shows that with increasing glycerol content, ΔH significantly increases (*p* < 0.05), however, no significant differences in T_p_ was observed among all the samples (*p* > 0.05). T_p_ is related to the thermal stability of starch, while ΔH represents the proportion of the ordered structure within the TPS with plasticizers that undergoes molecular rearrangement, accompanied by the formation of more ordered structures (54, 55). The S4/0 sample exhibited the highest ΔH, indicating a higher molecular order or a more stable crystal. This finding is consistent with the XRD results, indicating that S4/0 has the largest relative crystallinity. Glycerol can enter starch molecules and form new hydrogen bonds that promote the migration of starch chains and lead to the rearrangement and crystallization of starch molecules. Therefore, ΔH increased with increasing glycerol content. Generally, changes in the crystalline structure of starch particles can be expressed by the melting temperature (T_c_, T_o_). The increase in T_c_-T_o_ indicates that the crystallization heterogeneity of the TPS samples is more pronounced (56).

**Table 3:** Thermal properties of TPS samples plasticized with different proportions of glycerol and D-mannitol.

Samples	T_o_ (°C)	T_p_ (°C)	T_c_ (°C)	T_c_ - T_o_ (°C)	ΔH (J/g)
S4/0	52.33 ± 0.31^b^	1.36 ± 0.09^a^	63.70 ± 0.73^a^	11.38 ± 0.64^a^	3.58 ± 0.57^a^
S3/1	52.90 ± 0.29^a^	1.30 ± 0.11^a^	63.38 ± 1.50^ab^	10.48 ± 1.46^ab^	2.42 ± 0.34^b^
S2/2	53.15 ± 0.37^a^	1.23 ± 0.06^a^	62.73 ± 0.68^ab^	9.58 ± 0.90^bc^	2.23 ± 0.48^bc^
S1/3	53.40 ± 0.60^a^	1.19 ± 0.04^a^	62.75 ± 0.31^ab^	9.35 ± 0.48^bc^	1.65 ± 0.43^cd^
S0/4	53.45 ± 0.19^a^	1.15 ± 0.01^a^	61.85 ± 0.47^b^	8.40 ± 0.52^c^	1.45 ± 0.21^d^

Values are expressed as mean ± standard deviation of multiple replicated samples. Values in the same column with different letters (a–d) indicate significant differences (*p* < 0.05). ^ns^No significant difference (*p* > 0.05).

### 3.7 Analysis of thermal stability

**Figure 6:**
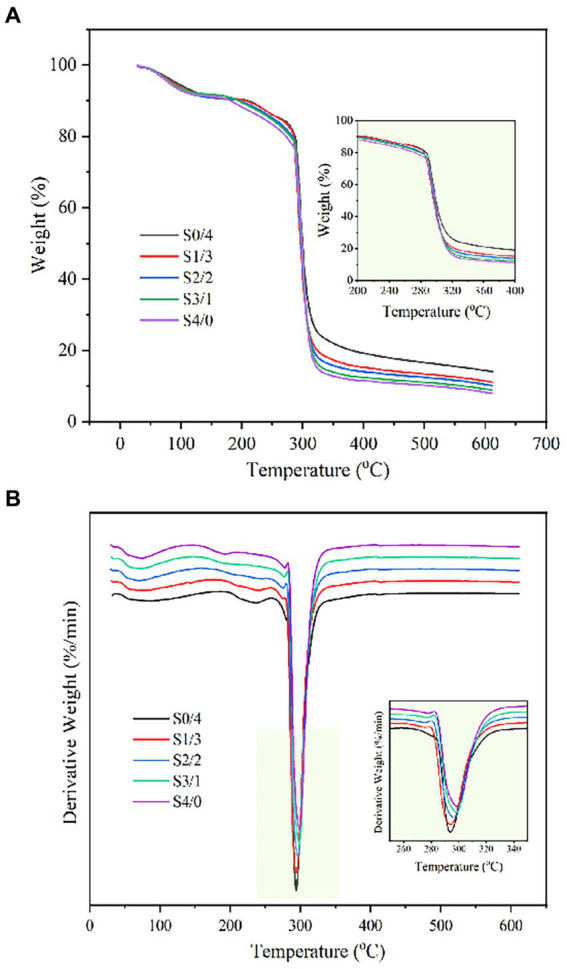
TG **(A)** and DTG **(B)** profiles of TPS samples plasticized with different proportions of glycerol and D-mannitol.

The TG and DTG curves of TPS samples: S4/0, S3/1, S2/2, S1/3, and S0/4 obtained by extrusion are shown in Figure 6. These curves can be used to analyze the thermal decomposition behavior and weight loss rate of the TPS samples. As shown in Figure 6A, the weightlessness process for all the TPS samples is divided into three stages. The mass loss in the first stage occurred between 30°C and 130°C and can be attributed to the loss of the bound water molecules from the TPS sample. The second stage occurred between 200°C and 400°C, where thermal decomposition of the plasticizers and thermal degradation of starch occurred. The weight loss above 400°C can be attributed to the reduction of ash (57).

Figure 6B shows that the maximum degradation temperature of the TPS samples increased from 293.97°C to 298.65°C with increasing glycerol content in the plasticizer blend, indicating that the increase in the glycerol content increased the thermal stability of TPS. Zhang et al. reported that the crystalline structure of starch is the main factor affecting its thermal stability (58). The crystalline region of starch is denser than its amorphous region and requires more energy for degradation. According to the measured XRD and infrared results, the higher the glycerol content, the higher the relative crystallinity and the higher the double helix and double helix order degree of the sample. Because the low molecular weight glycerol enters the starch molecular chains and binds to the hydroxyl groups, the fluidity of the starch chain is enhanced and a double helix is formed, thus increasing the thermal stability of TPS. However, the RC of TPS with a high D-mannitol content was lower, and the energy required for sample degradation was also lower (59). Gao et al. reported that the maximum degradation temperature of starch films decreased with an increase in the water/glycerol ratio (44). The results showed that the glycerol plasticized samples performed better thermal stability.

## 4 Conclusion

In this study, glycerol and/or D-mannitol were used as plasticizers to prepare TPS using the co-directional twin-screw extrusion mechanism under the same extrusion parameters. The effects of different plasticizer ratios on the properties of TPS were analyzed. The results showed that the RC, DO, and DD values of TPS increased with increasing glycerol content. TPS with a high glycerol content showed higher water resistance in aqueous systems. Glycerol is more likely to enter the starch interior for molecular interactions, promoting high mobility and high free volume of the starch chains. Therefore, increasing the glycerol content in the plasticizer blend results in higher gel stability, thermal stability, and molecular order of the TPS samples. The findings of the study indicate that glycerol, as a plasticizer, is more effective than D-mannitol in producing TPS with high thermal stability and water resistance, making it a suitable option for developing starch-based materials with similar characteristics. This study provides a theoretical basis for starch extrusion and plasticization in the preparation of TPS-based materials with specific properties.

## Data availability statement

The original contributions presented in the study are included in the article/supplementary material, further inquiries can be directed to the corresponding authors.

## Author contributions

XX: Methodology, Investigation, Formal analysis, Software, Writing – original draft. BW: Investigation, Writing – review & editing. WG: Investigation, Data curation. JS: Investigation, Formal analysis. JW: Investigation, Supervision. BCi: Resources, Conceptualization, Supervision, Writing – review & editing.
